# Role of Mobilome in Carbapenem Resistance

**DOI:** 10.3390/antibiotics15060542

**Published:** 2026-05-28

**Authors:** Laiba Hassan, Muhammad Ali Syed, Binghuai Lu, Jiankang Zhao, Bin Cao

**Affiliations:** 1 Department of Microbiology, The University of Haripur, Haripur 22620, Pakistan; laibahassan1996@gmail.com (L.H.); syedali@uoh.edu.pk (M.A.S.); 2 National Center for Respiratory Medicine, State Key Laboratory of Respiratory Health and Multimorbidity, New Cornerstone Science Laboratory, National Clinical Research Center for Respiratory Diseases, Institute of Respiratory Medicine, Chinese Academy of Medical Sciences, Department of Pulmonary and Critical Care Medicine, Center of Respiratory Medicine, China-Japan Friendship Hospital, Beijing 100029, China; zs25041@126.com; 3 Department of Pulmonary and Critical Care Medicine, Capital Medical University, Beijing 100069, China

**Keywords:** mobilome, carbapenem, plasmids, transposons, integrons

## Abstract

Growing resistance to carbapenem antibiotics is a major public health problem as these antibiotics are considered the last line of therapy for infections caused by multidrug-resistant (MDR) Gram-negative bacteria. The rapid emergence and dissemination of carbapenem-resistant bacterial strains are mainly due to horizontal gene transfer (HGT) within or between bacterial cells via the mobilome. The aim of this article is to discuss the role of mobile genetic elements (MGEs) that capture and disseminate resistance determinants of carbapenem antibiotics, as a comprehensive review integrating the combined role of plasmids, transposons and integrons. It attempts to systematically fill the gap by investigating the role of these MGEs in the acquisition, mobilization and dissemination of genes encoding carbapenemases across clinically important bacteria. Various types of plasmids such as IncF and IncH in *Klebsiella pneumoniae*, IncL/M in *Enterobacter cloacae*, IncX3 in *Escherichia coli* and IncA/C_2_ in *Salmonella enterica* carry important genes encoding carbapenemases. The rapid distribution of transposons among bacterial species is one of the main contributing factors in the dissemination of carbapenem-resistant isolates. Transposons including Tn*4401* carrying *bla*_KPC_ in *K. pneumoniae* and Tn*1721* carrying *bla*_KPC_ in *E. coli*; Tn*2006*, Tn*2007*, Tn*2008* and Tn*2009* carrying *bla*_OXA-23_ in *Acinetobacter baumannii*; Tn*1696* carrying *bla*_IMP-4_ in *Pseudomonas aeruginosa*; Tn*125* carrying *bla*_NDM_ in *E. coli*; and Tn*6306* carrying *bla*_IMI_ in *Raoultella ornithinolytica* encode different types of carbapenemases. Integrons mainly belonging to class 1 capture resistance determinants for metallo-carbapenemases such as NDM-, VIM-, SIM- and IMP-type enzymes in *P. aeruginosa*, *A. baumannii*, *K. pneumoniae* and *E. coli* and can promote the transcription and expression of these determinants. These findings are useful for understanding the genetics of carbapenem resistance and additional knowledge on MGEs may provide avenues for screening of resistance to these antibiotics in clinical settings.

## 1. Introduction

Multidrug-resistant (MDR) bacterial strains are a major health-associated problem all over the world. The evolutionary change encountered by both Gram-positive and Gram-negative bacteria has an important role in combating antimicrobial treatment for serious infections [1]. This is mainly achieved through the acquisition of pre-existing determinants of resistance from the gene pool of bacteria followed by amplification to selection response, meaning that bacterial populations increase rapidly under antibiotic pressure due to the presence of resistance genes. The capture, dissemination and accumulation of resistance genes is mainly due to mobile genetic elements (MGEs) such as transposons (Tn), integrons (Int), insertion sequences (ISs) and plasmids, commonly referred to as the mobilome [2]. These elements are responsible for horizontal gene transfer (HGT), which is defined as the exchange or transfer of genetic material from one bacterial cell to another as between donor cell and recipient respectively, which is further transferred to their offspring through cell division [3]. For example, in clinical settings transposable elements are the main source of the evolution of pathogenic antibiotic-resistant bacteria such as *Acinetobacter baumannii* (*A. baumannii*), which acquire resistance determinants by MGEs, making treatment complicated and challenging [4].

**Figure 1:**
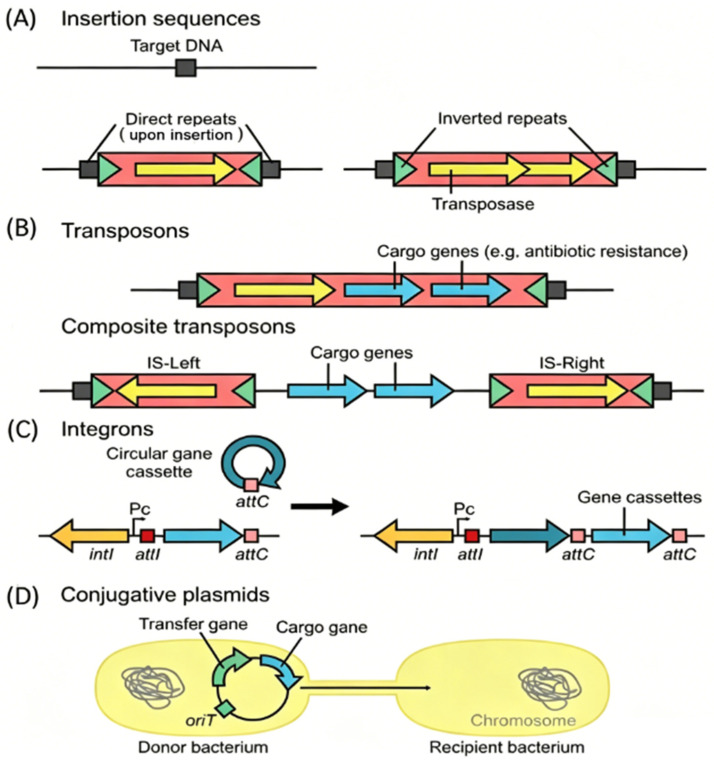
Structure of mobile genetic elements. (**A**) Insertion sequences: inverted repeats flank transposase gene in IS, which generates direct repeats after insertion into target DNA (also called transposition site duplication). (**B**) Transposons: same as IS but also contain cargo genes such as antibiotic resistance genes. Composite transposons: generated when IS elements flank cargo genes. (**C**) Integrons: contain *intI* gene which encode integrate gene cassettes at *att sites*. (**D**) Conjugative plasmids: origin of transfer (*oriT*) in conjugative plasmids transfer MGEs and cargo genes present in the plasmid from donor to recipient bacterium. **Note:** This figure is adapted from another research article [7] with permission.

Plasmids act as vectors for intercellular mobilization of resistance genes [2]. Transposons and insertion sequences are discrete segments of DNA that move resistance genes associated with them randomly and non-randomly to a new location in distinct or the same DNA molecules [5]. Integrons use the mechanism of site-specific recombination for moving genes of resistance between defined sites [3]. Interaction between different types of MGEs may result in rapid evolution of MDR strains which are even resistant to antibiotics considered as a last resort of treatment such as carbapenem and colistin in the era of antimicrobial chemotherapy [5,6]. Structures of different types of mobile genetic elements are shown on Figure 1.

This narrative review article focuses on presenting updated information on the important role of MGEs in the evolution and spread of carbapenem resistance genes. Since antibiotic resistance in general and MGEs in particular are very broad areas, the authors have specifically focused on the role of MGEs in carbapenem resistance. Literature searches were carried out by searching various key words such as mobile genetic elements + carbapenem resistance, integrons + carbapenem resistance, transposons + carbapenem resistance, insertion sequence + carbapenem resistance, etc. Search engines and databases such as Google, NCBI PubMed (https://pubmed.ncbi.nlm.nih.gov/), and Sciencedirect.com were searched to gather updated information. In addition to that the authors have also paid attention to the role of high-risk clones of certain bacterial species in the dissemination of MGEs and carbapenem resistance.

## 2. Carbapenems and Their Key Mechanism of Resistance

**Figure 2:**
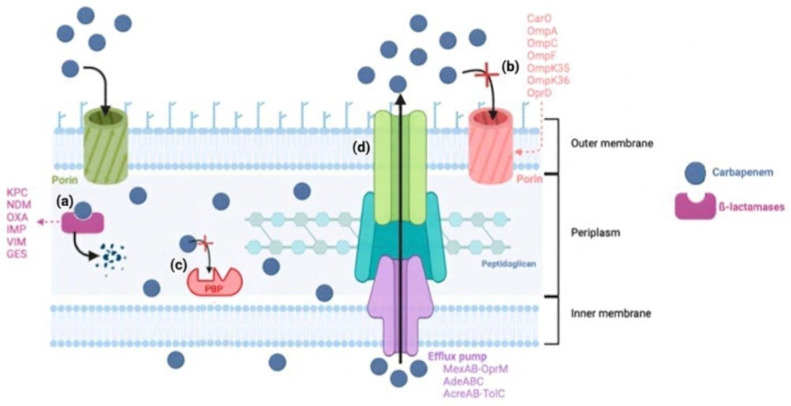
Mechanism of carbapenem resistance in Gram-negative bacteria. (**a**) Carbapenemase production by carbapenem-resistant bacteria, (**b**) decreased permeability of porins, (**c**) reduced binding of PBPs to the drug, (**d**) increased activity of efflux pumps (reproduced from [11]).

Carbapenems are β-lactam antibiotics with proven effectiveness in severe bacterial infections caused by extended spectrum β-lactamase (ESBL) producing, mostly against Gram-negative bacteria [8]. Carbapenem antibiotics have a broad spectrum of bactericidal activity. Examples of carbapenem antibiotics include meropenem, imipenem, ertapenem, doripenem, biapenem and panipenem which are used globally because of increasing resistance to cephalosporin antibiotics in the Enterobacterales bacteria [9]. Extended use of carbapenems have resulted in mediating carbapenem resistance among bacterial species. Resistance to carbapenems is acquired by mutational changes or acquisition of resistance genes through HGT [10]. Figure 2 shows the different mechanisms of carbapenem resistance.

**Figure 3:**
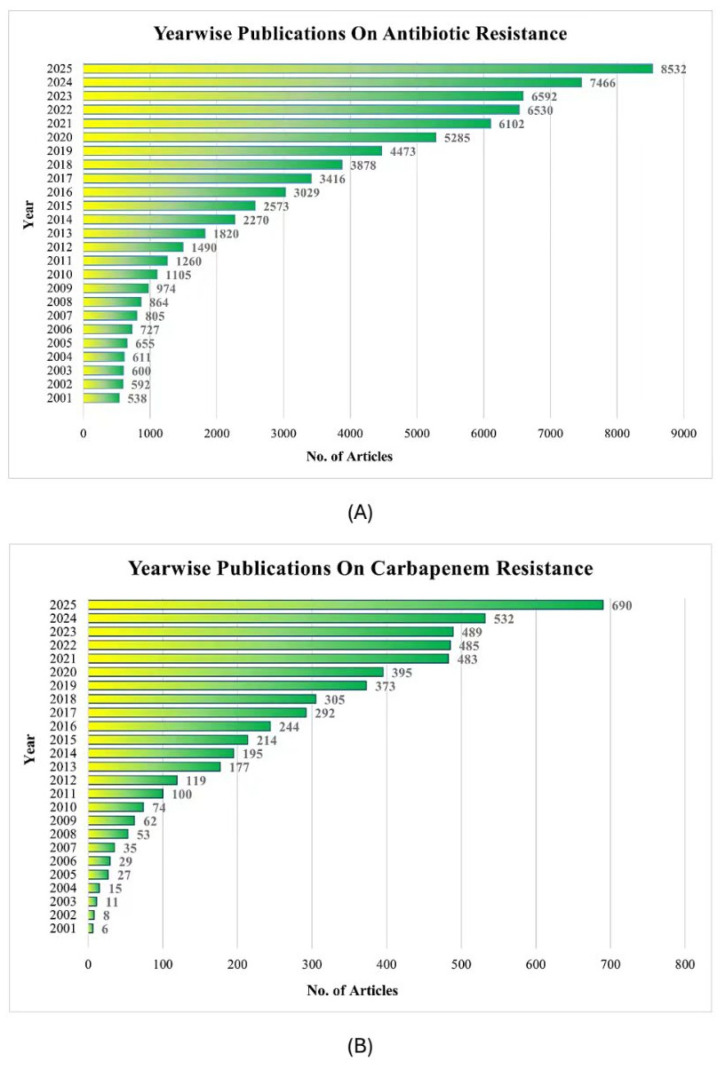
Number of published articles on antibiotic resistance (**A**) and carbapenem resistance (**B**). Searched using https://pubmed.ncbi.nlm.nih.gov on 22 March 2026. **Note:** This figure was generated by using MS Word and Excel.

Enzyme-mediated carbapenem resistance is the key resistance mechanism which occurs through production of carbapenemase enzymes hydrolyzing carbapenems as well as other b-lactam antibiotics. Carbapenemases are encoded by genes which transfer horizontally by MGEs such as transposons or plasmids. These MGEs may also carry other determinants of resistance [12]. A study conducted in Europe has shown the rapid spread of *K. pneumoniae* (*K. pneumoniae*) strain ST323 carrying a plasmid encoding gene for carbapenemase, i.e., *bla*_KPC-2_, into six different hospitals in Greece, potentially increasing the number of resistant strains [13]. Similarly, the emergence of hypervirulent carbapenemase-producing *K. pneumoniae* strains harbouring genes encoding either New Delhi MBL (NDM), *Klebsiella pneumoniae* carbapenemase (KPC), or Oxacillinase-48 (OXA-48) have been reported from Switzerland, representing a threat to public health all over the world [14]. There have been increasing reports of carbapenem resistance from all over the world in the last two decades, more notably from Asian countries (Figure 3).

### Carbapenemases and Their Classification

Carbapenemases are hydrolytic enzymes that break down the carbapenem drug, leading to antibiotic resistance in bacterial species. The carbapenemase genes mainly spread through mobilomes including plasmids, integrons and transposons [15].

B-lactamases are mainly classified into two major systems. The first one is the Ambler classification system which categorizes B-lactamases into classes A-D on the basis of their structure, including homology in amino acid sequences, which share characteristic protein folds within each class of enzymes and conserved catalytic domains; however, the substrate preference is all over the board, whereas the second one is the Bush-Jacoby-Medeiros classification system which categorizes B-lactamases on the basis of their functional properties, including the hydrolysis pattern of substrates and susceptibility pattern of inhibitors. For example, extended-spectrum beta-lactamases (ESBLs) including TEM, SHV, and CTX-M mainly hydrolyze extended-spectrum cephalosporins and are commonly inhibited by clavulanic acid. Carbapenemases such as NDM-, KPC-, IMP-, VIM-, and OXA-type enzymes hydrolyze carbapenems, whereas AmpC B-lactamases typically hydrolyze cephalosporins and are generally resistant to inhibitors of ẞ-lactamase such as clavulanic acid [16,17].

**Figure 4:**
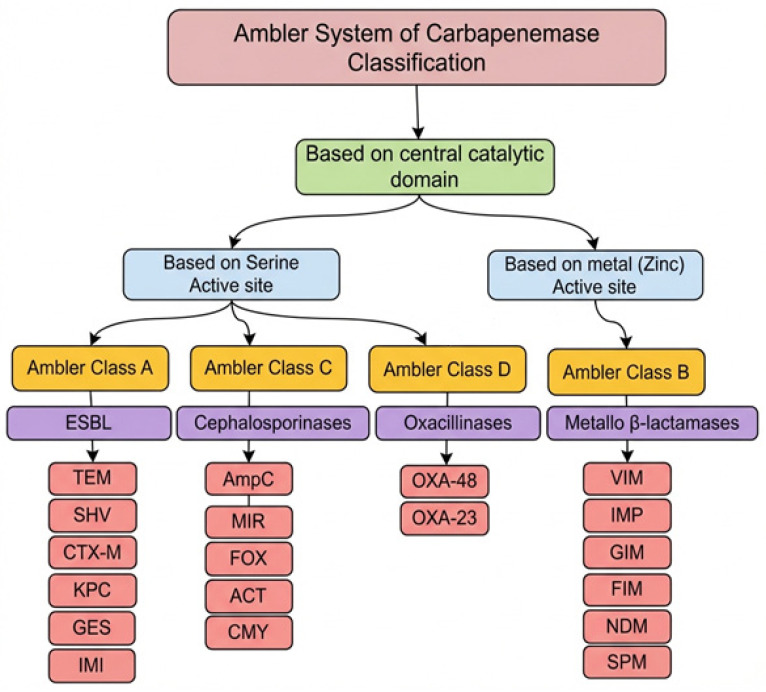
The Ambler system of beta-lactamase classification. **Note:** This figure is the authors’ own creation generated by using PowerPoint.

According to the Ambler classification system, carbapenemases are included in classes A, B, and D, while enzymes belonging to class C mainly hydrolyze cephalosporins (Figure 4). The active catalytic sites of enzymes belonging to the A, C, and D classes have serine, whereas class B enzymes have zinc in their active catalytic site [16]. ẞ-lactamases included in class A consist of very diverse and continuously increasing variants of enzyme identified across various species of bacteria and mobile genetic elements. However, most common enzymes that are epidemiologically and clinically important representative enzymes include KPC [18], SME (*Serratia marcescens* enzyme) [19], GES (Guiana extended-spectrum β-lactamase) [20], SHV (sulfhydryl variable lactamase) [21], SFC (*Serratia fonticola* carbapenemase) [22], and IMI/NMC-A (imipenemase/non-metallo-carbapenemase-A) [23]. The most representative enzyme of class A carbapenemase is the KPC-type enzyme, which is transmitted through plasmids, for example, the IncP-type plasmid [24]. The *bla*_KPC_ gene, responsible for KPC enzyme production, is mostly found on transposon Tn*4401* which facilitates the dissemination of the resistance gene mainly in *Enterobacteriacae* [25]. The overall structure of the KPC-type beta-lactamases is like other members of class A beta-lactamases. It consists of two sub-domains: one is mainly α helical, while the other has five beta sheets, flanked by α helices. The cleft found between two domains harbours an active site at S70 and E166, essential for catalysis. The active site is surrounded by three loops: the Ω loop containing R164 and D179 residues, the loop between α3 and α4 helices where W105 is located and a third loop that lies opposite to the Ω loop. Mutagenesis analysis has revealed that W105 plays a crucial role in ligand recognition by KPC-2 [26].

Class B β-lactamases are metallo-β-lactamases. Like class A, β-lactamases included in class B consist of very diverse and continuously increasing variants of enzymes identified across various species of bacteria and mobile genetic elements. However, most common enzymes that are epidemiologically and clinically important representative enzymes include Verona integrated-encoded MBL (VIM) [27], imipenemase (IMP) [28], Germany imipenemase (GIM) [29], Florence imipenemase (FIM) [30], NDM [31], and Sao Paulo MBL (SPM) [32]. The genes encoding these enzymes can reside on plasmids, transposons, integrons, the chromosome or other genetic elements. NDM encoded by the *bla*_NDM_ gene is mainly associated with MGEs such as plasmids and transposon Tn*125* that spread the resistance gene among various bacterial species [14]. Among MBLs, NDM is clinically one of the most significant families. The structure of NDM-type metallo-β-lactamases is composed of two domains; domain A consists mainly of β-sheets, while domain B possesses a mixture of α-helices and β-sheets. NDM is a divalent metal ion-dependent metallo-B-lactamase that mainly utilizes zinc ions for catalytic activity. The hydrolysis of the β-lactam ring involves activation of the water molecule facilitated by these metal ions which do not form any covalent intermediate. NDM-type enzymes exhibit a broad range of resistance to beta-lactam antibiotics, including carbapenems. These beta-lactamase enzymes are not inhibited by classical inhibitors but metal chelating agents such as EDTA can inhibit them [17,33].

Class C β-lactamases (AmpC-type enzymes) are generally not included in carbapenemases as they typically do not exhibit resistance to carbapenems [34]. However, a rare variant, CMY-10, has been reported to confer limited resistance which hydrolyzes carbapenem when combined with other mechanisms of resistance such as porin loss, so it has a supplementary role in conferring resistance [35].

β-lactamases belonging to class D are known as OXA enzymes. The OXA genes are frequently located on integrons and/or plasmids allowing wide dissemination of these genes [36]. For example, *bla*_OXA-23_ is associated with a higher rate of resistance to carbapenems in *A. baumannii* that is commonly found on MGEs [37]. OXA-type beta-lactamases have a typical two-domain structure. Their structure is composed of two domains; domain A consists of αhelices, while domain B possesses a mixture of αhelices and βsheets. The active site is situated in the cleft between the two domains. OXA-type ẞ-lactamases have conserved active-site residues consisting of Ser70 and the carboxylated Lys73 particularly. These residues form the core of the catalytic centre responsible for the hydrolysis of β-lactam, including carbapenems in certain variants of OXA. The acylation and deacylation steps of drug degradation take place in the active site of the enzyme [38,39,40]. The flow chart given in Figure 4 shows the classification of beta-lactamases.

## 3. Role of Plasmids in Carbapenem Resistance

Plasmids are transferred between bacteria horizontally by conjugation and transformation. Conjugative plasmids are the most important carriers of AMR in bacterial families like *Enterobacteriaceae* including some of the most important bacterial pathogens causing nosocomial infection [41,42]. Similarly, plasmids play a significant role in the dissemination of multiple genes of carbapenem resistance among bacterial cells [43,44]. An important example is plasmid pOXA-48 (carrying the carbapenemase gene *bla*_OXA-48_) in *K. pneumoniae* [45]. Carbapenem resistance in bacteria is mainly due to genes encoding hydrolyzing enzymes of carbapenem belonging to either class A carbapenemases, class B (metallo-ß-lactamases) or Ambler class D (oxacillinases) that are acquired horizontally [46] (Table 1).

The most serious resistance to carbapenems is caused by acquiring MBLs, as compared to other mechanisms of resistance, because these enzymes can hydrolyze almost all β-lactam drugs except monobactam [47]. MBLs belong to class B carbapenemases, and the genes encoding MBLs are located on mobile genetic elements such as integrons and plasmids and can easily spread among bacteria through HGT, causing serious problems in treating MDR bacteria [48].

Different types of carbapenemases such as NDM-1 in *K. pneumoniae*, (*E. coli*), *C. freundii*, *Enterobacter cloacae* (*E. cloacae*), *A. baumannii*, and *Pseudomonas aeruginosa* (*P. aeruginosa*); OXA-48 in *K. pneumoniae*, *Escherichia coli* (*E. coli*), *Enterobacter cloacae* (*E. cloacae*), *Citrobacter freundii* (*C. freundii*), *Serratia marcescens* (*S. marcescens*), and *Proteus mirabilis* (*P. mirabilis*); OXA-23 in *A. baumannii*, *Acinetobacter nosocomialis* (*A. nosocomialis*), and *Acinetobacter pittii* (*A. pittii*); KPC in *K. pneumoniae*, *E. coli*, *E. cloacae*, *S. marcescens*, and *P. aeruginosa*; IMP in *P. aeruginosa*, *A. baumannii*, *E. coli*, *K. pneumoniae*, and *Providencia* spp.; Australian imipenemase (AIM) in *P. aeruginosa* and *Pseudomonas fluorescens* (*P. fluorescens*); SPM in *P. aeruginosa*; Seoul imipenemase (SIM) in *A. baumannii* and *P. aeruginosa*; VIM in *P. aeruginosa*, *K. pneumoniae*, *E. coli*, and *Enterobacter cloacae*; GIM in *P. aeruginosa*; and Japan, Kyorin University Hospital imipenemase (KHM) in *C. freundii* and *Enterobacter* spp. have been identified all over the world [23,49]. In the following paragraphs examples of plasmids encoding some important types of carbapenemases are discussed.

### 3.1. Plasmids Encoding OXA-Type Carbapenemases

Carbapenem-hydrolyzing class D β-lactamases (CHDLs) comprise oxacillinases which is a highly diverse group, with more than 1400 OXA variants identified to date. Only a few variants of carbapenem-associated OXA families which are epidemiologically and clinically significant are highlighted. The predominant CHDLs include OXA-23-like, OXA-24/40-like, OXA-51-like, and OXA-58-like enzymes, which are associated with carbapenem resistance and have been identified in *Acinetobacter* species such as the *bla*_OXA-23_-like gene in *A. baumannii*, *Acinetobacter radioresistens* (*A. radioresistens*), *A. pittii*, and *A. johnsonii*; the *bla*_OXA-24_-like gene in *A. baumannii*, *A. pittii*, and *A. calcoaceticus*; the *bla*_OXA-51_-like gene in *A. baumannii*; and the *bla*_OXA-58_-like gene in *A. baumannii*, *A. pittii*, and *A. nosocomialis*. including *A. baumannii*, *A. pittii*, *A. johnsonii*, *A. radioresistens*, *A. johnsonii*, *A. nosocomialis* and *A. calcoaceticus*. The carbapenem-hydrolyzing activity of CHDLs is weak; however, a high level of resistance to carbapenem is acquired by the overexpression of these genes which is mostly driven by promotors found in their upstream ISs [50]. The *bla*_OXA-51_-like gene containing an IS*Aba1* element (IS*Aba1*-*bla*_OXA-51_-like gene) found upstream of this gene was initially located on a chromosome of isolates of *A. baumannii* [51,52]. A plasmid-borne IS*Aba1*-*bla*_OXA-51_-like gene has been identified in several carbapenem-resistant isolates of *A. baumannii*. The high level of resistance to carbapenem is occasionally caused by the overexpression of genes encoding CHDLs, mainly when the determinants of resistance are found on a plasmid, and this is possibly due to the plasmids with a higher copy number providing increased gene copies [53]. In addition to the spread of the *bla*_OXA-51_-like gene with upstream IS*Aba1* sequence by plasmids, the other extrinsic CHDL genes, such as the *bla*_OXA-58_-like, the *bla*_OXA-24_-like, and the *bla*_OXA-23_-like genes, were also apparently disseminated through plasmids in *A. baumanni* [54]. The dissemination of genes encoding CHDLs further complicate the control and treatment of infections caused by *A. baumannii* [53].

In Enterobacterales, OXA-48-like carbapenemases are another clinically significant subgroup of CHDLs. Among these, emerging variants such as OXA-484 have recently attracted attention due to their plasmid-mediated dissemination (pOXA-484), and association with multidrug resistance determinants, including qnrS1-mediated fluoroquinolone resistance in *E. coli*. However, OXA-484 represents an emerging OXA-48-like variant rather than as a uniquely important OXA enzyme [55]. Similarly, two plasmids harbouring the *bla*_OXA-48_ gene were identified in a research study conducted in Malawi. These plasmids, pEc_MW04_OXA in *E. coli* and pKv_MW05_OXA in *Klebsiella variicola* (*K. variicola*), share more than 99% similarity with the nucleotide sequence of the pandemic plasmid pOXA-48a, exhibiting worldwide spread across different bacterial species [56].

These findings highlight the significance of plasmids in the dissemination of OXA-type carbapenemases posing challenges for antibiotic therapy and infection control.

**Table 1:** A list of the important plasmids that are known to carry resistant genes for carbapenemases along with their associated class, incompatibility group, mechanism, and examples of host organisms.

Plasmid(s)	Class	Inc Group	Resistance Gene	Mechanism	Organism Examples	Reference
pKPC2	Class A (Serine carbapenemase)	IncFII, IncFIB,	*bla*_KPC-2_	Hydrolyzes carbapenems&other β-lactams	*K. pneumoniae*	[57]
pNDM-BJ01	Class B (MBL)	Various (IncA/C, IncF, IncL/M, IncX3)	*bla*_NDM-1_	Zinc-dependent hydrolysis of carbapenems	*E. coli*, *P. aeruginosa*, *A. baumannii*	[58]
pKpQIL	Class B (MBL	IncFIIK	*bla*_NDM-1_	Carbapenem hydrolysis	*K. pneumoniae*	[59]
p23045-NDM5	Class B (MBL)	IncHI2	*bla*_NDM-5_	Enhanced hydrolysis and resistance	*Salmonella enterica* (*S. enterica*)	[60]
pSXRJ10–250 K	Class B (MBL)	IncHI2	*bla*_NDM-5_	Enhanced hydrolysis and resistance	*Escherichia fergusonii* (*E. fergusonii*)	[61]
pPA166-2-MDR	Class B (MBL)	IncP-2	*bla*_IMP-45_	Carbapenem hydrolysis	*P. aeruginosa*	[62]
pIMP-4-BKP19	Class B (MBL)	IncN	*bla*_IMP-4_	Carbapenem hydrolysis	*K. pneumoniae*	[63]
pA52-OXA-72	Class D (Oxacillinase)	Un-classified	*bla*_OXA-72_	Carbapenem hydrolysis	*A. baumannii*	[64]

### 3.2. Plasmids Encoding NDM-Type Carbapenemases

This MBL type was first identified in two *E. coli* and *K. pneumoniae* strains from a Swedish patient admitted to a hospital in New Delhi, India. Recently, NDM-1-producing bacteria such as *K. pneumoniae*, *E. coli*, *Enterobacter cloacae*, *P. aeruginosa*, *A. baumannii*, *C. freundii*, *S. marcescens*, *S. enterica*, *Morganella morganii* (*M. morganii*) and *Providencia rettgeri* (*P. rettgeri*) have emerged and disseminated in various countries [65]. These bacteria which produce NDM are usually resistant to almost all antibiotic groups such as aminoglycosides, fluoroquinolones, and beta-lactams (mainly carbapenems), but show susceptibility to colistin and occasionally tigecycline [66]. The gene encoding NDM has been found on large plasmids such as pNDM-BJO1 in *Acinetobacter lwoffii* (*A. lwoffii*) and pNDM-SAL in *S. enterica* which can easily transfer and disseminate among bacteria causing a serious threat to public health [67]. Apart from this, *bla*_NDM_ genes are located on a variety of plasmid types, including IncF in *E. coli* and *K. pneumoniae*, IncH in *K. pneumoniae* and *S. enterica*, IncL/M in *E. cloacae* and *K. pneumoniae*, and IncX3 in *E. coli* and *K. pneumoniae*, as well as in a broad-host-range plasmid such as IncA/C_2_ in *S. enterica* and *E. coli* [68]. Plasmids such as IncHI2 and IncB/O/K/Z carrying *bla*_NDM-9_ have been identified in Carbapenem-resistant *Escherichia coli* (CREC) in 2025 in China [69].

#### 3.2.1. IncA/C2 Plasmids

IncA/C_2_ plasmids have a broad host range and usually carry NDM-1 in *K. pneumoniae*, *E. coli*, *S. enterica*, *E. cloacae*, *V. cholerae*, etc. [70]. The plasmid-encoding *bla*_NDM-1_ gene is found in the region of ARI-A, an island of resistance designated for IncA/C_2_ [71]. The resistance island has two parts, a Tn*1548*-like transposon and an IS*Aba125*-mediated composite transposon (Tn*125*) in pM214_A/C_2_ in *A. baumannii*, *E. coli*, and *K. pneumoniae* as designated in a previous study [72]. For example, IncA/C2 is associated with *bla*_NDM-1_ as identified in *K. pneumoniae*, resistant to carbapenems, also carries other resistance genes, and reported to be transferable to other bacterial species such as *E. coli*, *S. enterica*, *E. cloacae*, and *V. cholerae* [73].

#### 3.2.2. IncX3 Plasmids

IncX3 plasmids encode different variants of NDM by beta-lactamase genes such as *bla*_NDM-4_ in *E. coli*, *bla*_NDM-5_ in *E. coli* and *K. pneumoniae*, and *bla*_NDM-7_ in *E. coli* [74,75,76]. Generally, the IncX3 plasmids do not contain antimicrobial resistance genes other than *bla*_NDM,_ which plays an important role in the spread and evolution of the *bla*_NDM_ gene [75]. As IncX3 plasmids encode distinct variants of NDM, this suggests that these variants are most likely to have evolved through substitutions of nucleotides within the IncX3 plasmid [74]. This process for selection of different variants might be due to stronger β-lactamase activity of NDM-4, -5, and -7 exhibiting higher resistance than NDM-1 [77]. A study found that IncX3 is the most common single replicon plasmid carrying *bla*_NDM-5_ in the US and East Asia, mainly associated with the ST48, ST167 and ST410 sequence types of *E. coli* [78].

#### 3.2.3. IncH Plasmids

The IncHI1B plasmid was identified in *K. pneumoniae* carrying *bla*_NDM-1_ as revealed by comprehensive analysis of its genome. The plasmid was also carrying other heavy metal and antibiotic resistance genes, highlighting its adaptability and complexity [79].

#### 3.2.4. IncFII Plasmids

The most predominant plasmid type containing *bla*_NDM_ was IncFII in *E. coli*, *K. pneumoniae*, *C. freundii*, and *Enterobacter cloacae.* In addition to genes for other β-lactamases, these plasmids also carry resistance genes for various antibiotics including sulfonamides, macrolides, trimethoprim, and aminoglycosides which contrast with IncX3 plasmids [80]. Also, the genetic structure of the IncFII plasmids is comparatively diverse, containing various insertion/deletion sequences and clear genetic mobilization traces through IS*26* [72]. IncF is a multi-replicon plasmid frequently carrying *bla*_NDM-5_ in *E. coli* that may be a conjugative or non-conjugative plasmid. The conjugative type was mainly found in *E. coli* ST167, contributing to worldwide dissemination including India, East Asia, the US and many European countries [78]. A novel multi-replicon IncFIB-HI1B plasmid harbouring *bla*_NDM-5_ has been characterized in Enterobacteriales in a study conducted in Argentina. The *bla*_NDM-5_ gene was found along with other antimicrobial resistance markers such as bla_CTX-M-15_ in an antimicrobial resistance island [81].

### 3.3. Plasmids Encoding KPC-Type Carbapenemases

KPC is a β-lactamase which can hydrolyze all b-lactam antibiotics such as penicillins, cephalosporins, monobactams, and carbapenems resulting in worse therapeutic outcomes by leaving fewer therapeutic options for infected patients [82]. The reports of *bla*_KPC_ gene detection, mostly *K. pneumoniae* multilocus sequence type 258 (ST258) carried by IncF type plasmid has been detected worldwide which shows that clonal spread of this resistant lineage is the main factor in the dissemination of *bla*_KPC_ [83,84,85]. A novel ST1076 sequence type of *P. aeruginosa* producing KPC-3 reported in China facilitates clonal dissemination of KPC-mediated carbapenem resistance. The *bla*_KPC-3_ gene is carried by a unique mega plasmid, and IncP-2. *bla*_KPC_ has also been detected in other lineages of *K. pneumoniae*, as well as other *Enterobacteriaceae* species, indicating that HGT of *bla*_KPC_ plays an important role in the dissemination of AMR [86]. *bla*_KPC_ is most often detected on conjugative plasmids in multiple species or strains such as *K. pneumoniae*, *E. coli*, *S. marcescens*, *P. aeruginosa* and *E. cloacae* providing a likely mechanism for HGT [87].

A study conducted in Israel detected the presence of a 105 kb plasmid encoding KPC-3- and TEM-1 in an extremely drug-resistant (XDR) *K. pneumoniae* strain. This strain emerged in 2006 causing an outbreak all over the region. This plasmid was termed as *pKpQIL* which was identified in all isolates during 2006–2008 belonging to an extensive β-lactam resistance clone which was highly epidemic. The carbapenem resistance in these *K. pneumoniae* isolates was mainly due to this single self-transmissible plasmid [88,89].

### 3.4. Plasmids Encoding VIM-Type Carbapenemases

The VIM enzyme is one of the most identified MBLs, which are predominant in Europe and Asia. Although many variants of VIM have been identified all over the world, among which VIM-1-like and VIM-2-like are the predominant plasmid-associated enzymes and are clinically and epidemiologically significant subgroups. VIM-1 has been identified in *P. aeruginosa*, *K. pneumoniae*, *E. coli*, *E. cloacae*, and *Pseudomonas putida* (*P. putida*), whereas VIM-2 has been reported in *P. aeruginosa*, *A. baumannii*, *K. pneumoniae*, and *Enterobacter* spp. [90,91]. Genes encoding VIM-1 carbapenemase have been detected on plasmids. Two plasmids (pAMBL1 and pAMBL2) carrying blaVIM-1 have been recovered from clinical isolates of *P. aeruginosa*. pAMBL1 is a 26,440 bp plasmid which carries a replication protein belonging to the RepA_C family. The plasmid pAMBL1 is like the pAX22 plasmid from *Achromobacter xylosoxidans* (*A. xylosoxidans*) and the pKLC102 and pNOR-2000 plasmids from *P. aeruginosa*, which also carry genes for VIM-type carbapenemases. The plasmid pAMBL2 is 24,133 bp with a replication protein belonging to the Rep_3 family. This plasmid shows a higher degree of similarity to a fragment of the plasmid pPC9 bearing blaVIM-1 from *P. putida*. The plasmid pAMBL2 confers a high level of carbapenem resistance as compared to pAMBL1 by carrying the blaVIM-1 cassette with three copies in class 1 integron In70 [92].

Similarly, plasmids such as pP6VIM-11 and pPOta2VIM-11 carrying blaVIM-11 genes have been identified in clinical isolates of *P. aeruginosa*. These plasmids belong to the IncP-1β group. The gene was found to be located on the class-1 integron which was flanked by different insertion sequences exhibiting its key role in integrating resistance genes into plasmids [93].

### 3.5. Plasmids Encoding IMP-Type Carbapenemases

Imipenemase enzymes, discovered first in Japan in *P. aeruginosa*, are the key drivers of carbapenem resistance in bacteria. Imipenemases are endemic in Asia and Australia, the genes of which are found on diverse types of plasmids facilitating the spread of imipenemases. Molecular epidemiological analysis of bacterial genomes suggests that the IncHI2A, IncC and IncN plasmids collectively account for 56.0% of all plasmid types carrying *bla*_IMP_ genes, each carrying four, two and five IMP variants [94,95]. These *bla*_IMP_ carrying Inc types were found to be endemic in Asia and Australia, while sporadic in Europe [94,96]. A study conducted in UK hospitals on the characterization of imipenemase-enzyme-producing bacteria at local hospitals revealed that 10 out of 18 imipenemases produced by *Enterobacterales* strains (mostly belonging to *K. pneumoniae* and *E. clocae*) were IMP-1, carried on IncN plasmid and in In1763 class 1 integron [96]. IncN group plasmids are a class of plasmids, that may be transferred to other bacterial communities including members of *Enterobacteriales* through transconjugation [97]. A recent study carried out in China on WGS analysis of 61 imipenemase (IMP)-producing strains of *K. pneumoniae* reported that the *bla*_IMP_ gene was harboured by IncN-IncR1 plasmid in 10 out of 61 strains [98]. All strains belonged to different sequence types (STs). Nevertheless, the volume of available data is still insufficient.

A recent study from China by Qu et al. (2026) reported the co-existence of *bla*_NDM-1_ and *bla*_IMP-4_ in transposon Tn*AS3* of plasmid IncHI5 harboured by carbapenem-resistant *Raoultella ornithinolytica* (*R. ornithinolytica*) [99]. IncHI5 plasmids are a group of large plasmids (~200 kb) carrying a number of resistance genes that have a broad host range including enterobacterales and some other species of Gram-negative bacteria [100].

## 4. Role of Transposons in Carbapenem Resistance

Transposons are mobile sequences of DNA in the genome that can jump into various locations; therefore, they are termed “jumping genes” and are grouped into MGEs [101,102]. A study conducted in 2021 showed that transposons can also cause resistance to carbapenems by preventing the entry of antibiotic through outer membrane porins by disrupting their genes. This disruption is caused by transposon insertion in porin genes at high frequency leading to reduced susceptibility of carbapenem in bacterial cells. This mechanism of transposon action underscores its role in carbapenem resistance by rapid adaptations to its genetics under increased antibiotic pressure [103]. Several transposon types carry important genes causing resistance to carbapenems and play an important role in the dissemination of these genes.

### 4.1. Transposon Tn4401 Carrying bla_KPC_ Genes

*bla*_KPC_ is commonly present on the Tn*4401* transposon, a 10 kb mobile genetic element belonging to the Tn3 family. This element contains two ISs, such as ISKpn6 and ISKpn7, and is flanked by inverted repeats of 38 bp [104]. There are five isoforms of Tn*4401*, a–e, which are differentiated by upstream deletions from the KPC gene [105]. The Tn*4401* transposons have been detected in various chromosomal insertions and in plasmids [106]. *bla*_KPC-2_ associated with Tn*4401*a located on plasmid pKPC-484 and *bla*_KPC-3_ associated with Tn*4401*b located on plasmid p34399-43.500kb were each detected in two patients having no obvious connection of epidemiology. *bla*_KPC-2_-associated Tn*4401*a has been found in *K. pneumoniae*, *E. coli*, and *E. cloacae* and *bla*_KPC-3_-associated Tn*4401*b found in *K. pneumoniae* (major host), *P. aeruginosa*, and *Enterobacter* spp. The IncI-type plasmid, pBK15692, was first detected in a strain of *K. pneumoniae* from a patient in a New Jersey hospital in 2005 carrying a KPC-3 gene associated with the Tn*4401*b transposon [104] (Table 2).

### 4.2. Transposons Tn2006, Tn2007, Tn2008 and Tn2009 Carrying bla_OXA_ Genes

One of the most important carbapenem resistance genes is *bla*_OXA-23_ in *A. baumannii*, which is harboured by transposons. The mobilization of *bla*_OXA-23_ occurs by Tn*2006*, Tn*2007*, Tn*2008* and Tn*2009* transposons in *A. baumannii* causing the spread of the *bla*_OXA-23_ gene by a transposon-mediated mechanism instead of clonal spreading of resistance genes [107]. Three transposons Tn*2006*, Tn*2008* and Tn*2009* have *IS* upstream of *bla*_OXA-23,_ while in Tn*2007*, a copy of IS*Aba4* is located upstream to *bla*_OXA-23_ [108,109]. In Tn*2006*, two IS copies of IS*Aba1* surround *bla*_OXA-23_ at both ends in opposite directions. Tn*2008* is the same as Tn*2006*, but the second IS*Aba1* copy is absent in it. Many studies have reported that currently Tn*2006* is the most common carbapenem resistance determinant, which mainly spreads among isolates of *A. baumannii* [108].

**Table 2:** List of the important transposons that are known to carry resistant genes for carbapenem along with their associated class or family, mechanism, and examples of host organisms.

Transposon	Transposon Class/Family	Resistance Gene	Type of Carbapenemase	Organism Examples	References
Tn*3000*	IS26-based composite	*bla*_NDM_	Metallo-β-lactamase	*K. pneumoniae*, *E. coli*	[110]
Tn*125*	ISAba125-associated	*bla*_NDM-1_	Metallo-β-lactamase	*E. coli*, *A. baumannii*	[111]
Tn*4401*	Tn3-like	*bla*_KPC-2_	Serine β-lactamase	*E. coli*, *K. pneumoniae*	[112]
Tn*4401*a/b/c	Tn3-like	*bla*_KPC-3_	Serine β-lactamase	*K. pneumoniae* ST258	[113]
Tn*402*	Class 1 integron platform	*bla*_VIM-1_	Metallo-β-lactamase	*P. aeruginosa*, *Enterobacteriaceae*	[114]
Tn*2008*	IS26-based composite	*bla*_OXA-23,_ *bla*_OXA-72_	Serine β-lactamase	*A. baumannii*	[115]
Tn*2006*	Composite transposon (ISAbal)	*bla*_OXA-23_	Serine β-lactamase	*A. johnsonii* *A. baumannii*	[116]
Tn*7493*	Tn*1403*-like transposon	*bla*_OXA-10_	Serine β-lactamase	*P. asiatica*	[117]
Tn1999.7	Composite transposon (IS1999)	*bla*_OXA-48_	Serine β-lactamase	*E. coli*	[118]

A study reported association of *bla*_OXA-23_ with the Tn*2006* transposon in *A. baumannii* (major host), *A. nosocomialis* and *A. pittii.* A *bla_OXA-23_* variant was identified in the Tn*2008*-like transposon (submitted as Tn*2008VAR* with GenBank accession number KT852972) from GC2 isolates of *A. baumannii*. This transposon is flanked by target site duplications of 9 bp, comprising IS*Aba1* and IS*Aba33*, a novel insertion sequence for transposition as mobilization of *bla*_OXA-23_ carrying transposons occurs via insertion sequences. IS*Aba1* insertions were indicated to be chromosomal by the assembly data. The copy number of *bla*_OXA-23_ carried by the Tn*2006* transposon was low within GC1 isolates carrying AbaR4 on a Aci6-type plasmid within which a single copy of IS*Aba1* was found [119]. OXA-23 carbapenemase production is among the most common causes for increasing carbapenem resistance among bacterial species, primarily *Acinetobacter baumannii*, which represents its main epidemiological reservoir [120]. For example, a study conducted in 2022 in southwestern Iran isolated 170 *A. baumannii* from clinical samples, which were resistant to carbapenems. Genomic analysis indicated the high prevalence of *bla*_OXA-23_ in *A. baumannii* (41.7%) and *bla*_OXA-24_ (55.3%) carried by Tn*2009* in *A. baumannii* and *A. pittii* and IS*Aba1* highlighting the role of transposable elements in spreading carbapenem resistance [121]. Although sporadic reports have shown the presence of *bla*_OXA-23_ in non-*Acinetobacter* species such as *Proteus mirabilis* and a few other Enterobacterales, such reports are rare and do not show sustained dissemination in these organisms [122].

### 4.3. Transposon Tn1696 Carrying bla_IMP-4_

The main contributor involved in dissemination of the carbapenem resistance *bla*_IMP-4_ gene is the Tn*1696* transposon in *E. coli*, *K. pneumoniae*, *C. freundii*, *E. cloacae*, and *P. aeruginosa* and it is surrounded by IS*5075* elements. This transposon is inserted into the class I integron In2 to constitute its main structure. Tn*1696* transposons belong to the family of Tn*3* transposons, which are usually reported in *E. cloacae* and *K. pneumoniae*. Tn*1696* transposons may gain increasing attention in the future for their role in the dissemination of the carbapenem *bla*_IMP-4_ gene [123].

### 4.4. Transposon Tn1721 Carrying bla_KPC-2_

It is reported that the Tn*1721* transposon is the main transposon carrying *bla*_KPC-2_ in bacterial species of the *Enterobacteriaceae* family such as *E. coli*, *K. pneumoniae*, *Enterobacter cloacae*, *S. marcescens* and *C. freundii* [124]. The *bla*_KPC-2_ gene for carbapenemase is located between insertion sequences IS*Kpn8* and IS*Kpn6*, forming the region “IS*Kpn8-bla*_KPC-2_-IS*Kpn6*,” which is downstream inserted in Tn*3* transposase to constitute a Tn*1721*-based transposon. This transposon can mediate the *bla*_KPC-2_ gene transmission between various bacterial species; therefore, the Tn*1721* transposon may be further transmitted to other bacterial strains [123].

### 4.5. Transposon Tn125 Carrying bla_NDM-1_

Many studies reported that composite transposon Tn*125* is formed by the presence of the *bla*_NDM_ gene between two IS copies such as the IS*Aba125* elements which carry *bla*_NDM-1_. Transposon Tn*125* carrying the *bla*_NDM_ gene has been found in *A. baumannii*, *K. pneumoniae*, *E. coli*, *P. aeruginosa*, and *Raoultella planticola* (*R. planticola*). In Tn*125*, a hybrid promoter sequence is present at -35 in the IS*Aba125* element which is responsible for the *bla*_NDM_ gene expression. Tn*125* has been identified in *P. aeruginosa* and *Enterobacteriaceae* [125]. The transposon Tn*125* carries the *bla*_NDM-1_ carbapenemase gene located between *bla*_MBL_ and insertion sequence IS*Aba125* forming the main structure of this transposon. The gene structure “IS*Aba125-bla*_NDM-1_-*bla*_MBL_” is mainly stable in the transposon Tn*125*. Almost all of the carbapenemases encoded by *bla*_NDM-1_ genes reported worldwide are found in this structure. The transposon Tn*125* is mainly located on a conjugated plasmid due to which the bla_NDM-1_ gene transmission becomes easier [126]. It is considered that the origin of Tn*125* is *A. baumannii* which was disseminated in this strain [127]. Later, a mobile sequence element was inserted in this transposon, e.g., the ISCR insertion sequence, as a result of which the novel Tn*125* transposon was particularly disseminated in *Enterobacteriaceae* species, especially *E. coli* and *K. pneumoniae* [128]. Tn*125* was also identified in the *Raoultella* spp. showing that the resistance genes in these species might have been acquired from bacterial isolates of *Enterobacteriaceae* [123]. Transposon Tn*125* and transposon Tn*3000* flanked by insertion sequence IS*26* are composite transposons which play an important role in the global dissemination of *bla*_NDM_ causing carbapenem resistance [129]. In a recent study, the Tn*125* transposon carrying *bla*_NDM-1_ was identified in *K. pneumoniae* along with the co-existence of *bla*_IMP-4_ located on a class 1 integrons suggesting the role of mobilomes in transferring multiple genes of carbapenem resistance together [130].

### 4.6. Transposon Tn6306 Carrying bla_IMI_

A retrospective study conducted in China identified a novel Tn*6306* composite transposon harbouring *bla*_IMI_ in an IMI-2-producing strain of *E. coli* RJ18 and the IMI-3-producing strain of (*R. ornithinolytica*) RJ46C. These strains were resistant to carbapenems. The Tn*6306* transposon was totally inverted between plasmids pGA45 and pRJ46C indicating that this novel transposon may play important roles in the mobilization and dissemination of *bla*_IMI_ genes [131]. The *bla*_IMI_ identification in *E. coli* and (*R. ornithinolytica*) indicated the diversity of disseminating carbapenemases in bacterial species of *Enterobacteriaceae* between the environment and hospitals in China [102].

Other carbapenemase-encoding genes have been found to be associated with various other transposons such as transposon Tn*1999* carrying the *bla*_OXA-48_ gene in *E. coli*, *K. pneumoniae*, and *E. cloacae* [132].

A novel transposon, Tn*7722*, was identified in a recent study conducted in 2024 from NDM-1-producing *K. pneumoniae* strains resistant to carbapenem. The Tn*7722* transposon was a composite transposon relating to the IS*6* family and measuring 16,246 bp. It was found on IncR and IncF plasmids. This transposon also carried resistance genes such as *aph* (3′)-VI and *qnr*S1 for aminoglycoside and fluoroquinolone resistance, respectively. The presence of this transposon in bacterial cells from different continents underscores its role in the global dissemination of carbapenem resistance mediated by *bla*_NDM-1_ [133].

### 4.7. Role of Transposons in the Emergence and Spread of Carbapenem-Resistant High-Risk Clones

High-risk clones of Gram-negative bacteria are MDR or XDR strains, such as *P. aeruginosa* ST235 and *E. coli* ST131, which spread globally due to high tenacity and flexibility in accumulating resistance genes. The emergence and spread of high-risk clones of bacteria in general and Gram-negative bacteria in particular present a serious global challenge. Genetic plasticity and metabolic diversity of some bacterial species (e.g., *E. coli*, *P. aeruginosa*) enable them to become MDR and XDR and facilitate their spread in different environments [134]. High-risk clones of *P. aeruginosa* belonging to the ST235 and ST654 carrying *bla*_KPC-2_ located in the Tn*4401b* transposon have been identified from Latin America [135]. As discussed in the preceding sections, transposons play a key role in the spread of carbapenem resistance genes. Likewise, their role in the emergence of high-risk clones of different Gram-negative bacterial species is obvious. For example, the MDR high-risk clone of ST235 of *P. aeruginosa* was reported to carry Tn*4401b*-*bla*_KPC-2_ from a study in Columbia [136]. Similarly, a recent study from China reported a novel transposon Tn*6485f* encoding *bla*_IMP-45_ and *bla*_AFM-1_ carried by XDR *P. aeruginosa* ST463 [137]. Another study reported a high-risk clone of XDR *P. aeruginosa* ST111 carrying Tn*4401b* associated with *bla*_KPC-2_ [138].

Several high-risk clones of E. coli are also associated with widespread carbapenem resistance. For instance, E. coli ST410 is a global clone showing resistance to fluoroquinolones, cephalosporins and carbapenems [139]. A recent study from Greece reported MDR *E. coli* ST410 carrying *bla*_KPC-2_ in the Tn4401 transposon of IncX3 plasmid [140]. *E. coli* ST131 is also a global high-risk clone. A study conducted in China reported that *bla*_KPC-2_ was located in Tn3 transposon. A study from Brazil reported high-risk clone *E. coli* ST167 carrying *bla*_OXA-181_ flanked by Tn3-like IS3000 composite transposons [141].

*K. pnuemoniae* is a medically significant bacterial species causing a range of health-care-associated infections. Several sequence types such as ST258, ST11, ST437, ST307, ST147, etc., belong to high-risk clones [142]. Transposons have been a key factor in the dissemination of carbapenem resistance genes in these strains. For examples, a study from Ecuador found that *K. pneumoniae* ST258 harboured the *bla*_KPC-2_ gene in Tn*4401*a inserted in transferable pKpQIL-like IncFII_K2_ plasmid [143]. Similarly, *K. pneumoniae* ST307 is a high-risk clone widespread in European countries. A recent study from China reported *K. pneumoniae* ST307 carrying *bla*_IMP-38_ in Tn6363 located on IncHI5 plasmid [144]. Another study on genetic analysis of an XDR high-risk *K. pneumoniae* ST11 clone from Brazil reports carriage of *bla*_KPC-2_ in Tn*4401*a [145]. Such findings suggest a key role played by Tn*4401* in the spread of the *bla*_KPC-2_ gene in high-risk clones as well as other strains.

Carbapenem-resistant *Acinetobacter baumannii* (*A. baumannii*) infections are of great concern in health care settings, with the death toll surpassing 40% in critically ill patients. Here too certain high-risk clones exist that spread highly resistant infections and have global distribution. One such extremely concerning global clone is ST2, notorious for carbapenem resistance and carriage of *bla*_Oxa-23_. A study from China on *A. baumannii* ST2 reported its presence on Tn*2006* transposon [116]. *A. baumannii* ST25 is another high-risk clone belonging to International Clone-7 (IC 7). According to a Nigerian study on high-risk clone *A. baumannii* ST25, *bla*_OXA-23_ was carried by Tn2006 [111]. Taking together, Tn*2006* seems to be a major type of transposon associated with the occurrence and spread of high-risk clones in *A. baumannii*.

## 5. Role of Integrons in Carbapenem Resistance

Integrons are genetic elements of dsDNA defined by the presence of a gene, which encodes the enzyme integrase called *IntI*. Integrons are classified as MGEs and are located in bacterial chromosomes, plasmids and transposons. Integrons carry the resistance genes that are located within the gene cassette in integons. The most successful method for the spread of resistance genes is the horizontal transfer of resistance integrons, which results in the emergence of MDR strains [146]. Integrons capture resistance determinants of antibiotics and can promote the transcription and expression of these determinants [147]. Recently, it has been recognized that class 1 and class 2 integrons play an important role in acquiring and disseminating carbapenem resistance genes such as *bla*_IMP_, mainly among Gram-negative bacteria. For example, in imipenem-resistant *A. baumannii*, class 1 integrons carry *bla*_IMP_-5; similarly in *P. aeruginosa* and *E. cloacae*, *bla*_IMP_ is commonly found in class 1 integron [148]. VIM and IMP carbapenemases are commonly encoded on class I integrons containing an integrase gene, *intlI*, linked with a transposase *tnp*A found upstream of this integron. *bla*_VIM_ is carried by class 1 integron in *P. aeruginosa* and *K. pneumoniae.* These metallo-β-lactamase genes are mostly associated with aminoglycoside resistance genes (*aad*A1, *aac*A4 and/or *aad*B), class D β-lactamases (*bla*_OXA_ genes), antiseptic resistance (qacΔG), and chloramphenicol resistance (*cat*B) [149]. The class 1 integrons belong to mobile integrons which are classified into five groups and always carried by plasmids; however, superintegrons are large integrons (approximately 126 kb) which have been found on chromosomes [150]. A genomic study revealed that genes encoding metallo-β-lactamase are responsible for carbapenem resistance and its spread through gene exchange mediated by integrons [151]. Metallo-β-lactamases such as VIM-, SIM- and IMP-type enzymes are reported to be important carbapenemases among clinical isolates of *A. baumannii*, the genes for which are carried by class 1 integrons. The studies of Da Silva et al. detected the class 1 integron carrying *bla*_IMP-5_ in *A. baumannii* resistant to imipenem [152]. Class 1 integrons carrying *bla*_SIM-1_ for carbapenemase among *A. baumannii* clinical isolates has been reported from Korea [153]. In another study, class 1 integrons conferring resistance to carbapenems have been identified in *A. baumannii* carrying gene cassettes (*bla*_NDM-1_, *bla*_OXA-23_ and *bla*_OXA-5_) that encode carbapenemases for carbapenem degradation, thereby impairing antibiotic effectiveness and facilitating resistance dissemination by HGT [154]. Class I integrons are associated with the dissemination of resistance genes of antibiotics in *Enterobacter* species along with their widespread role of carrying *bla*_VIM_ and *bla*_IMP_ genes [155].

*bla*_NDM-1_ was found on a 4.3 kb region in a plasmid associated with the 4.8 kb complex class I integron. The same *bla*_NDM-1_ genetic structure, as identified in the *Enterobacteriaceae*, has also been detected in *P. aeruginosa*, where the similar genetic structure is found in the variable region carrying the insertion sequence ISCR1 complex class I integron inserted into the chromosome of bacteria [124]. The relation between molecular forms of VIM-carrying *P. aeruginosa* and the genetic context was assessed in another study conducted during 2011 in Thessaly. Forty-six percent of MDR VIM-producing *P. aeruginosa* belonging to ST111 and ST235 clusters were reported previously to carry *bla*_VIM-2_ and *bla*_VIM-4_ enzymes encoding gene cassettes. Novel VIM-2-encoding integrons were detected among sporadic isolates. A novel *P. aeruginosa* ST (ST1457) isolate carrying a VIM-17-encoding integron was also detected in a study. These findings show the high prevalence of *P. aeruginosa* resistant to carbapenems producing VIM-type enzymes along with their growing evolution [156]. Similarly, a study conducted in Iran has shown 64% of class 1 integrons and 20% of class 2 integrons in carbapenem resistance *P. aeruginosa*. These integrons carry gene cassettes such as IMP and VIM metallo-β-lactamases which degrade carbapenems showing their correlation with MDR [18].

A study conducted in Nigeria reported the presence of carbapenemase genes such as *bla*_VIM-5_ in the *P. putida* group (*P. guariconensis* and *P. plecoglossicida*) isolated from environmental sources. Whole genome sequencing revealed the presence of *bla*_VIM-5_ in three novel Tn*402*-like structures of class 1 integron carrying the cassette arrays *bla*_VIM-5_|*aadB*|*tnpA*|*bla*_PSE-1_|*smr2*|*tnpA*, *aadB*|*bla*_VIM-5_|*aadB*|*bla*_PSE-1_, and *aadB*|*bla*_VIM-5_|*bla*_PSE-1_ [149].

*Klebsiella* species also contain genes for MBLs such as IMP-type and VIM carbapenemases associated with class I integrons. Isolates of *K. pneumoniae* carried the *intI1* gene (36.6%) in the study results conducted in 2011 and 2015 in which the *bla*_VIM-1_ gene cassette prevalence was 30% [150,151]. In another study on plasmids of *K. pneumoniae*, researchers identified integrons associated with IS*26* elements. These elements carried gene cassettes for carbapenemases such as KPC-2 enabling bacteria to survive in the presence of carbapenem antibiotics [152].

**Figure 5:**
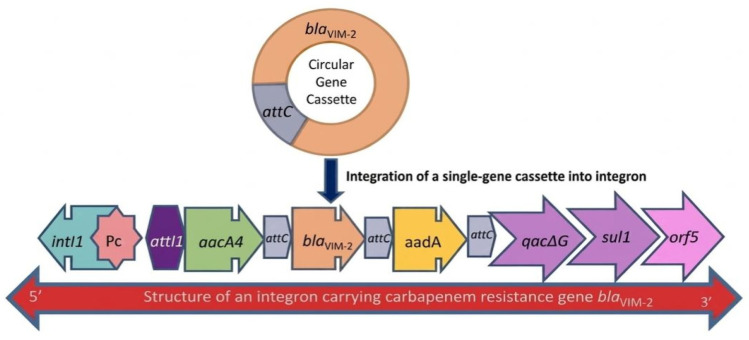
The structure of an integron and its role in the spread of the antibiotic resistance gene. Integrons comprise integrases encoding gene *intI* that catalyzes the recombination process between *attI* recombination site (on the integron) and *attC* site (on the circular gene cassette), resulting in the integration of multiple different gene cassettes, including the carbapenem resistance gene, sequentially to create a tandem array of a gene cassette containing hundreds of genes. A promotor, Pc, encoded by the integron expresses the integrated genes. **Note:** Figure developed using MS PowerPoint.

The class 1 integrons associated with MBLs play an important role in the dissemination of these resistance genes in nosocomial infections caused by pathogens. Integron- and plasmid-borne variants of IMP have also been detected in many other enteric bacteria [153]. Carbapenemase genes such as *bla_KPC_*, *bla_NDM_*, and *bla_VIM_* have been found mostly in Int-1 but few in Int-2 from *E. coli* isolated from patients with UTI [154]. The structure of integron carrying a carbapenem resistance gene is shown in Figure 5. Thus, integrons play an important role in the assembly and spreading of carbapenem resistance genes among *Enterobacteriaceae*. Integrons enable these bacterial species to resist antibiotic pressure exerted by carbapenems by expressing carbapenemase genes [155].

## 6. Carbapenem Resistance in Food, Livestock and Aquaculture

As discussed previously, increasing bacterial resistance to carbapenems, a valuable class of antibiotics against Gram-negative bacteria, is alarming for health care professionals globally. A rational approach of investigation and management of ABR is the *One Health* strategy. Collaboration and cooperation between different sectors aimed at integrating health involving plants, animals, humans and the environment can give synergistic output. The One Health approach is considered an essential strategy to deal with emerging infectious diseases as well as the spread of antibiotic resistance [156,157].

The presence of MDR bacteria in food and livestock, agriculture and associated environment is alarming. Since there has been an overall increase in the use of carbapenem, resistance and the rate of antibiotic resistance have been increasing; the food and agriculture sectors also seem to be badly impacted. European countries usually restrict the use of carbapenems in livestock, while other countries do not have any regulations. The results of studies conducted across the globe reveal the presence of carbapenem resistance from food, dairy, and livestock as well as associated products with varying degree of prevalence. Despite the ban on the use of carbapenems, there is a relatively low prevalence of CRB being reported from livestock, food and dairy products [157,158,159,160,161,162].

Mobile genetic elements play a key in driving resistance to carbapenems in the food sector. For example, conjugative plasmids such as IncA/C, IncF, IncHI, IncL/M and IncX spread genes encoding carbapenemases among food pathogens including *K. pneumoniae*, *E. coli*, *A. baumannii* and *S. Enterica* in animal food and products using the antibiotic in agriculture, increasing their persistence [2,163]. Class 1 integrons further enhance the dissemination of carbapenem resistance by capturing multiple gene cassettes and promoting co-selection when integrated in transposons or plasmids [164]. In addition, insertion sequences and transposons such as ISAba1 and *Tn*4401 accelerate the expression and mobilization of carbapenemase genes like *bla_KPC_* and *bla_OXA_*, enhancing dissemination and stability in food-associated bacteria [2,161].

The presence of ARB strains of bacterial pathogens such as *Salmonella*, *Klebsiella*, *E. coli*, *Shigella* spp., etc., in water and the food supply chain poses a significant threat to human health, since food is a major necessity of human life and the food supply chain may involve travel to different cities or even countries. Pathogenic bacteria constitute a small portion of the overall microbial population in food, livestock and agricultural products, since most of the microbial population is non-pathogenic. Nevertheless, the presence of resistant bacteria may disseminate antibiotic resistance genes to the non-pathogenic population through horizontal gene transfer. For instance, the bacteria of normal flora of fish, poultry and domestic cattle may acquire ABR and hence become a major source of spread to humans through animal contact and food consumption [160,161].

In the livestock sector, mobile genetic elements (MGEs) mainly spread carbapenem resistance through conjugative plasmids, integrons, transposons and insertion sequences, enabling horizontal gene transfer of carbapenemase (e.g., *bla*_NDM_, *bla*_KPC_, *bla*_OXA48_-like) among Gram-negative microbiota in animal gut [2,163]. Broad-host-range plasmids such as IncA/C, IncF, and IncX3 act as major gene transfer vehicles for interspecies [164], while elements like Tn4401, Tn125, IS26, and ISAba125 promote the integration and mobilization of resistance determinants [2]. Although carbapenems are rarely used in animals, co-selection by using other antimicrobials stabilize these MGEs, allowing the spread and persistence of genes through food supply, manure, and the environment [165], thereby linking livestock reservoirs with human infections under a One Health framework [163].

In recent years, several studies have been carried out on the detection and molecular characterization of carbapenem-resistant bacteria (CRB) from cattle, poultry, sea food and their products. For instance, a study conducted in Texas USA on cattle feces, detected several carbapenem-resistant strains. They identified *A. baumannii* producing *bla*_OX-497_ and *Pseudomonas* spp. producing conserved domains of *bla*_IMI_ and *bla*_OXA_, indicating the role of these genes in horizontal gene transfer among bacteria in cattle normal flora [166].

Meat and dairy products like raw milk may also contribute to dissemination of ABR. Raw milk is still consumed in several countries of the world; hence it may be a significant source of transmission of AMR. A study conducted in Turkey, reported detection of *bla*_NDM_, *bla*_KPC_, *bla*_VIM_ and bla_OXA-48_ genes from *E. coli* and *K. pneuminiae* strains isolated from raw milk samples, underscoring the importance of continued surveillance of ABR in food and dairy products as well as pasteurization [167].

Poultry farms, live poultry markets and their environment appear to be a major source of transmission of antibiotic-resistant strains, underscoring the importance of the One Health aspect of investigation. Results of several recent studies conducted on poultry and poultry environment have published alarming results with high prevalence of carbapenem resistance genes such as *bla***_NDM_** in the isolated bacterial strains. A mention-worthy study recently conducted in China reported up to 72% detection of *bla*_NDM_ genes in poultry feces at Live Poultry markets in bacterial species including *E. coli*, *Enterobacter cloacae*, *Proteus mirablis* (*P. mirablis*), *K. pneumoniae*, etc. Poultry meat may be an important means of transmission of ABR strains, since it is one of the most consumed food types all over the world, equally both in developed as well as developed countries. Antibiotics [if present] may persist in the poultry meat even after washing and continue exerting selection pressure on bacteria [168]. A study conducted in India on detection and characterization of CRB in poultry meat reported presence 36.8% carbapenem resistance in different bacterial species, i.e., *Morganella morganii* (*M. morganii*), *Providencia alcalifaciens* (*P. alcalifaciens*), *Stenotrophomonas pavanii* (*S. pavanii*), *Klebsiella aerogenes* (*K. aerogenes*), *P. mirabilis*, *Providencia huaxiensis* (*P. huaxiensis*), *Proteus terrae* (*P. terrae*), *Stenotrophomonas maltophilia* (*S. maltophilia*) and *K. pneumoniae.* They have been able to detect *bla*_OXA-48_ like *bla*_IMI_ *bla*_KPC_ genes from these strains with varying degree of occurrence [169].

Water reservoirs offer a key source of antibiotic ARB, especially in the towns and communities where sewerage water is not appropriately decontaminated and broken drinking water supply lines are contaminated with it. In addition to this, fresh water and marine sources offer an ideal environment for the transmission of pathogens to human population, whereas favourable habitat of these environments facilitate horizontal gene transfer of ARGs. Furthermore, use of recycled human wastewater poses a significant heath challenge nowadays. Quality of treatment performance and post treatment practices need to be strictly monitored in order to ensure overall human and environmental safety. Disposal of wastewater into freshwater reservoirs is a common practice in less developed countries, risking the overall health of aquatic life and also transmission of contaminated food through food chains including fish [170]. A few recent articles have reported detection of CRB from fish and other sea food from both fresh water and marine sources.

Carbapenem resistance in aquaculture disseminate mainly via MGEs that enable horizontal transfer of carbapenemase genes among aquatic pathogenic bacteria. Broad-host-range conjugative plasmids, mainly IncA/C, IncHI2, IncF, IncL/M, and IncX3, carry carbapenemase genes such as *bla*_NDM-1_, *bla*_NDM-5_, *bla*_KPC-2_, *bla*_OXA-48_-like which facilitate rapid spread in fish farming environments under antimicrobial pressure. Transposons such as *Tn*_125_ (*bla*_NDM_), *Tn*_4401_ (*bla*_KPC_), and derivatives of *Tn*_21_/*Tn*_3_ help in mobilizing these genes between plasmids and chromosomes, increasing their stability and dissemination in aquaculture [171,172]. Class 1 integrons further integrate resistance gene cassettes (e.g., aadA, qnr, and blaVIM-associated arrays), promoting multidrug resistance in dense aquatic microbial communities such as *Aeromonas* spp. and *Shiwanella* spp. [173,174]. An interesting study in India carried out on detection and analysis of carbapenem-resistant *E. coli* identified high-risk clones of ST167 and ST361 from freshwater fish samples carrying *bla*_NDM-5_. It may be mentioned here that Indian sub-continent is considered hot region for NDM, with higher prevalence rate of carbapenem resistance [175].

In recent decades aqua culture has been developed significantly to meet consumer demands. Aqua culture accounts for 50% of sea food production. Sea food contamination with pathogenic microorganisms, including CRB, has also been reported. A US study reported the presence of *bla*_IMI-2_ in *Enterobacter* spp. and *bla*_NDM-1_ in *Acinetobacter* spp. from retail fish samples [176].

## 7. Future Perspective

Despite significant advancements and ongoing dedicated efforts in understanding the vital role of mobile genetic elements in the evolution and dissemination of carbapenem resistance, the dynamic interplay between bacterial hosts, MGEs and diverse environments of clinical settings as well non-clinical habitats continue to pose a continues challenge. Keeping in view the seriousness of the issue, future research must shift from retrospective characterization to a prospective, predictive, and therapeutic framework, alongside applying advanced analytical approaches [177,178].

Whole genome sequencing (WGS) has become a cornerstone in infectious disease research and surveillance. The field is now poised to extend its focus from descriptive genomics to predictive modelling. Multi-omics approaches integrated with the power of computation and bioinformatics may enable prediction of the likelihood of transmission of particular MGEs to high-risk clones or other members of bacterial species based on the sequence features. In addition to this, investigation of MGE transfer within complex communities (e.g., human gut) using sequence-based techniques, combined with artificial intelligence/machine learning (AI/ML), may identify the hotspots of gene transfer and predict how antibiotic use may shape up the mobilome of bacteria in a given habitat or a person and accelerate the rate of emergence of drug resistance [178,179].

Since most of the research studies on drug resistance are carried out under in vitro settings, which fail to recapitulate the complexity of the in vivo conditions, in vivo gut models may be employed to systematically understand the transmission dynamics of MGEs during clinical treatment, co-infection with bacteriophages or in the presence of immune response. Furthermore, applying advanced technologies such as long-read sequencing (Nanopore sequencing) of the bacterial strains directly from the patient’s sample would better help us understand the mechanism of gene transfer, without the biases of laboratory culturing.

The One Health approach is becoming a cornerstone of infectious disease surveillance research. The mobilization and spread of carbapenem resistance genes occur across human, animal and environmental sectors. It has now become inevitable to monitor drug resistance by phenotypic methods or detection of ARGs by molecular methods such as polymerase chain reaction (PCR). Surveillance and intervention research related to carbapenem resistance must involve the One Health framework. There is a need of unified, global surveillance networks with standard operating procedures and willingness to dedicate efforts to combat antibiotic resistance. This all requires standardized protocols to carry out metagenomic analyses of water, food, agricultural runoff, and wildlife to identify environmental reservoirs and transmission corridors that link to clinical outbreaks [180].

## 8. Conclusions

Increasing antibiotic resistance levels among bacterial pathogens constitute a major challenge to global health, with carbapenem resistance being a specifically concerning problem as these antibiotics are the last line of therapy for serious infections. The MGEs such as plasmids, transposons and integrons form a complex mobilome which plays a significant role in the horizontal gene transfer of carbapenem resistance determinants among various bacterial species, underpinning the intimidating adaptive potential to carbapenem resistance. MGEs are one of the significant contributors in the dissemination of antibiotic resistance that can be shared by various strains and species of bacterial genera, making them one of the largest concerns for public health. MGEs can facilitate the rapid spread of carbapenemase genes within bacterial species and geographical areas due to rapid diffusion of plasmids, integrons and transposons. These elements play an important role in bacterial evolution and adaptation including antibiotic resistance which results in the emergence of carbapenem-resistant bacteria. Understanding the role of MGEs in the dissemination of carbapenem resistance and more knowledge on the mobilome is useful as it may open ways for screening of resistance to these antibiotics in clinical settings.

## Data Availability

No new data were created or analyzed in this study. All sources of information have been cited in the article. Data sharing is not applicable to this article.

## Abbreviations

MGE	Mobile genetic element
HGT	Horizontal genen transfer
Tn	Transposon
IS	Insertion sequence
ESBL	Extended-spectrum beta-lactamase
PBP	Penicillin binding protein
MBL	Metallo beta-lactamase
FDA	Food and drug administration
LPS	Lipopolysaccharide
CHDLs	Carbapenem hydrolyzing class-D beta-lactamase.
PetN	Phosphoethanol amine
XDR	Extensively drug resistance
PDR	Pan drug resistance
KPC	*Klebsiella pneumoniae* carbapenemase
SME	*Serratia marcescens* enzyme
GES	Guiana extended-spectrum β-lactamase
SHV	Sulfhydryl variable lactamase
SFC	*Serratia fonticola* carbapenemase
IMI/NMC-A	Imipenemase/non-metallo-carbapenemase-A
VIM	Verona integrated-encoded MBL
IMP	Imipenemase
GIM	Germany imipenemase
FIM	Florence imipenemase
NDM	New Delhi MBL
SPM	Sao Paulo MBL
OXA	Oxacillinases
